# The association between marital quality and diabetes mellitus: A systematic review

**DOI:** 10.1002/hsr2.1106

**Published:** 2023-02-07

**Authors:** Mohsen Rastkar, Erfan Jalalifar

**Affiliations:** ^1^ Students' Scientific Research Center, Tehran University of Medical Sciences Tehran Iran; ^2^ Student Research Committee, Tabriz University of Medical Sciences Tabriz Iran

**Keywords:** Diabetes Mellitus, Diabetes Mellitus, Type 1, Diabetes Mellitus, Type 2, marital quality, marital relationship quality

## Abstract

**Background and Aims:**

Marital relationship and its quality are among the major psychological factors affecting the multiple aspects of a person's health status. Chronic diseases are also among the factors that affect various aspects of the lives of millions of people including their marital quality status. One of the most important underlying chronic diseases is diabetes. Since the correlation between diabetes mellitus and marital quality has been neglected, this systematic review, as the first one, aims to investigate the association between marital quality and diabetes mellitus.

**Methods:**

A comprehensive search was conducted among three databases (Medline, Scopus, and Web of Science) until September 2021, which resulted in 189 articles. After assessing the studies based on the inclusion criteria, 14 studies were included.

**Results:**

The included studies were divided into two general groups. The first group consisted of 3 articles examining the effect of factors related to diabetes on marital quality, and the second group included 11 articles studying the effect of marital quality on diabetes and its factors. In general, the articles investigating the impact of diabetes‐related factors on marital quality showed that diabetes has negative impacts on levels of marital quality. Also, the articles investigating the impact of marital quality on diabetes‐related factors, showed that higher marital quality is associated with a lower risk of developing diabetes, a better quality of life in patients with diabetes, and better adherence to diabetes care regimen. The results regarding diabetes management were conflicting. Gender was mentioned as an important modulator in some of the investigated relationships.

**Conclusion:**

Marital quality remarkably influences diabetes‐related factors and is itself affected by the condition resulting from diabetes in individuals with diabetes mellitus. However, further studies are required due to the limited number of studies investigating this correlation.

## 1 INTRODUCTION

Marital relationship and its quality are among the major psychological factors that affect the multiple aspects of a person's health status. Marital quality is defined as a way of determining the overall quality of marriage by several positive and negative characteristics.^1^ There are numerous questionnaires designed for the measurement of marital quality such as the Dyadic Adjustment Scale (DAS), which is the most widely used in the studies.^2^ Furthermore, marital quality is investigated based on its multiple aspects including positive dimensions^3^ like marital satisfaction, adjustment, intimacy, positive supportive interaction, marital support, constructive communication, and enjoyment as well as negative dimensions such as marital stress, marital risk, marital tension, marital strain, and negative unsupportive interactions. The marital relationship quality is considered an important factor related to the quality of life which affects various aspects of health. Some studies have shown that marital quality is directly related to physical health.^4^ One of the important factors that should be considered about psychological factors is their relationship and association with chronic diseases. Diabetes is considered a significant global health challenge due to lifestyle changes in recent years. According to a comprehensive report, the Global prevalence of diabetes mellitus was approximately 4.4% (415 million people) in 2015 and it is predicted to reach 10.8% or 642 million people by 2040.^5^ Considering the alarming prevalence of diabetes mellitus, this disease has attracted the attention of many researchers in this field.^6^ Diabetes mellitus, as a metabolic disease, is identified by hyperglycemia which can cause damage to different organs and may result in several complications. Regarding the characteristics of the disease, diabetes mellitus can be categorized into different types, including type 1 and type 2 diabetes.^7^ Diabetes mellitus may lead to other health‐related outcomes, including adverse effects on psychological factors such as the increase in depression among patients with diabetes.^8^

As mentioned, psychological factors are among the variables that affect different aspects of people's lives.^1^ In society, one of the groups that must always keep their lives under severe control to maintain their health is people with chronic diseases.^9^, ^10^, ^11^ Diabetes, as one of the most common chronic diseases,^5^ has profound effects on different dimensions of people's lives.^8^, ^12^, ^13^ In people with chronic diseases such as diabetes, it is important to know the factors that can affect their quality of life and help them manage their disease better. Marital quality, as well as the relationship between partners, is a factor that most people face in their lives. Therefore, its impact on people's life is undeniable.^4^ In this situation, it is necessary to investigate the impact of marital quality on various dimensions related to diabetes. Also, due to the conditions it imposes on a person's life, diabetes can affect the marital quality of people,^13^, ^14^, ^15^ and during this process, diabetes may affect itself recursively. In this situation, it is necessary to investigate the mutual impact of marital quality and diabetes, so that related risk factors can be identified and the possibility of effective interventions to improve the quality of life of people with diabetes can be provided.

The influences of diabetes mellitus on the quality of the marital relationship are discussed by several studies.^13^, ^14^, ^15^ However, this relationship has been overlooked by researchers and should be investigated in more studies. Also, marital quality can have major effects on various diabetes‐related factors, such as disease development, diabetes management, quality of life, and adherence to diabetes care regimen.^16^, ^17^, ^18^, ^19^, ^20^, ^21^, ^22^, ^23^, ^24^, ^25^, ^26^ In a situation where this issue has never been systematically investigated, new studies in this field are designed and implemented without a strong background, and therefore it is not possible to make the most of the potential of the obtained results. In this situation, this study, as the first systematic review to investigate the association between marital quality and diabetes mellitus, aims to become a guide for future studies to choose their objectives more accurately.

## 2 METHOD

### 2.1 Search strategy

A comprehensive search was conducted in Medline, Scopus, and Web of Science databases to identify and review the literature on marital quality and diabetes mellitus. A search strategy was designed based on the combination of two groups of marital quality and diabetes mellitus keywords. Keywords were chosen by searching MeSH terms, reviewing related articles, and consulting with researchers. The search strategy for each of these databases is provided in the Supplorting Information: File.

### 2.2 Inclusion/exclusion criteria

The articles were included according to these criteria: (1) the studies investigating the relationship between marital quality and diabetes mellitus; (2) the article was published in English

The exclusion criteria were as follows: (1) studies discussing the relationship between marital quality and other factors in patients with diabetes which were not related to the disease (2) reviews, letters, conference papers, and editorials

**Figure 1:**
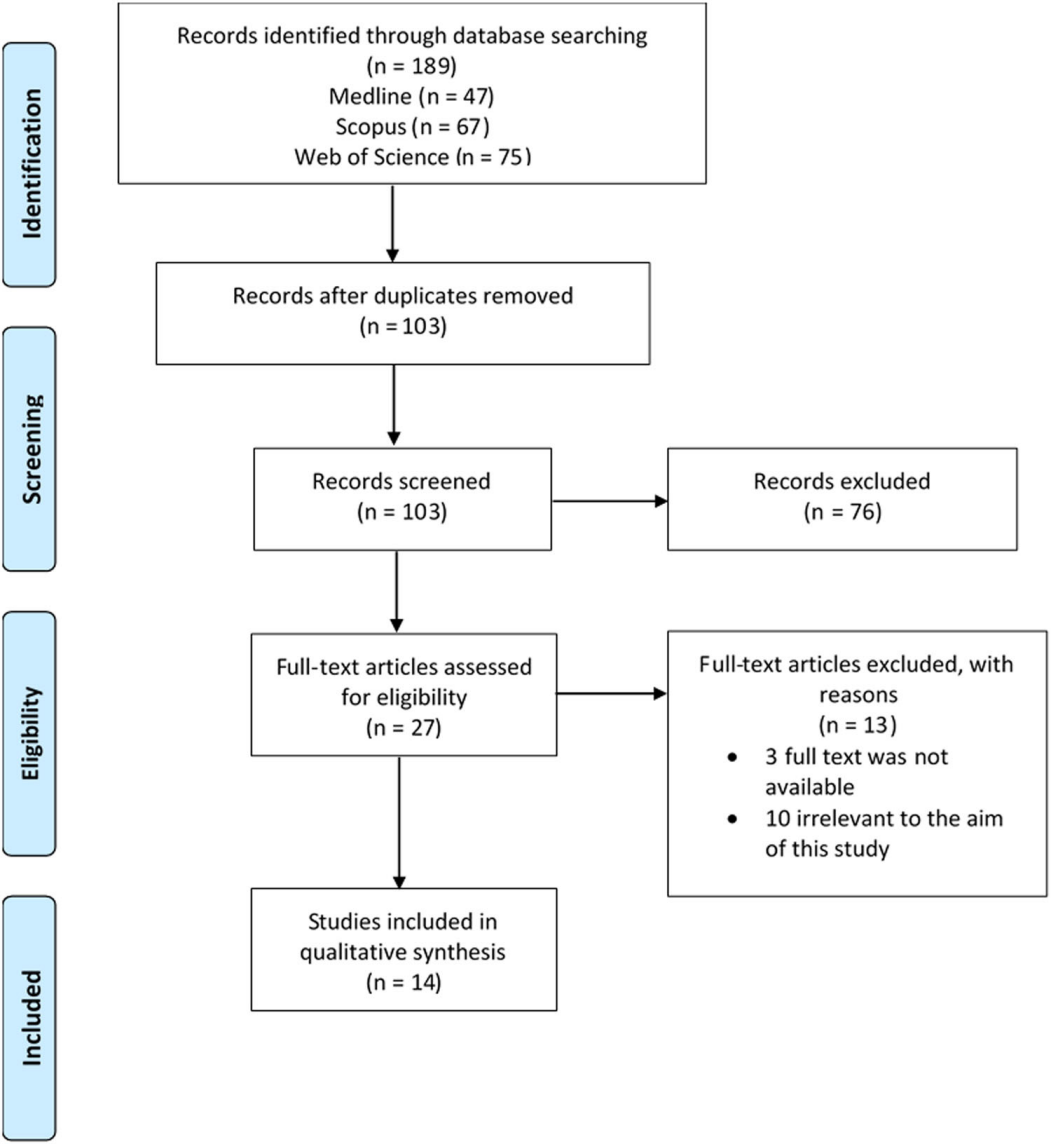
The diagram of literature search.

Authors (M. R. and E. J.) independently searched the databases and screened the title and abstracts of the articles based on the inclusion/exclusion criteria. After the first screening, potentially eligible articles were screened by two reviewers (M. R. and E. J.) based on reviewing the full text according to inclusion/exclusion criteria. The mentioned steps were performed while the two authors were blinded to each other. The disagreements on articles were resolved by discussion between authors. After performing the above steps and according to the inclusion/exclusion criteria, 14 studies were included in this systematic review. Figure 1 illustrates the diagram of the literature search.

### 2.3 Risk of bias assessment

We used the Newcastle‐Ottawa scale^27^ to evaluate the cohort studies. This scale consists of three groups: selection, comparability, and outcome. A cohort study can get a maximum of 1 score for each question of selection and outcome, and a maximum of 2 scores for comparability. Thus, a study can get a maximum of 9 scores from the Newcastle‐Ottawa scale. Table 1 shows the result of the qualitative analysis of cohort studies.

**Table 1:** Qualitative analysis of cohort studies.

Study	Selection				Compatibility		Outcome			Total score
Trief et al.^24^		*	*	*	*	*	*			6
Whisman et al.^26^	*	*	*	*	*	*	*			7
Liu et al.^18^	*	*	*	*	*	*	*	*		8
Roberson et al.^21^	*	*	*	*	*	*	*	*		8
Trief et al.^25^		*	*	*	*	*	*			6
Trief et al.^23^		*	*	*	*	*	*			6

Also, an adapted version of this scale was used for evaluating cross‐sectional studies which include three groups of selection (maximum of 5 scores), comparability (maximum of 2 scores), and outcome (maximum of 3 scores). Table 2 shows the result of the qualitative analysis of cross‐sectional studies.

**Table 2:** Qualitative analysis of cross‐sectional studies.

Study	Selection					Compatibility		Outcome			Total score
Enzlin et al.^13^	*		*	*		*	*	*		*	7
Schreiner‐Engel et al.^15^	*		*	*		*	*	*		*	7
Dadgari et al.^16^	*		*	*		*		*		*	6
Fisher et al.^17^	*			*		*		*		*	5
Martire et al.^19^			*	*		*		*		*	5
Trief et al.^20^			*	*		*		*	*	*	6
Pieper et al.^14^				*		*		*		*	4
Naqvi et al.^20^	*	*	*	*		*	*	*		*	8

### 2.4 Data extraction

The required data were extracted by two independent researchers and the findings were reported based on PRISMA.^28^ A predefined table was used for extracting data including first author name, publication date, country, study design, sample number, age and gender characteristics, marital parameters and measurement tool, diabetes‐related parameters, and results (Table 3).

**Table 3:** Characteristics of included studies.

Reference	Study design and duration (*N* = total sample number)	Place of study	Mean age (SD) or age range	Gender distribution	Marital parameters (Factors related to the quality of marriage that were investigated and the related measurement tools)	Diabetes‐related parameters (Related diabetes factors that were investigated in the study)	Significant results
Trief et al.^24^	Prospective cohort study over 2 years *N* = 78	US	Mean age of 45.7 (11.4)	42% male 58% female	Marital adjustment (the Spaniar Dyadic Adjustment Scale [DAS]) Marital intimacy (Personal Assessment of Intimacy in Relationships Inventory [PAIR])	Adherence to diabetes care regimen	↑Marital quality and intimacy: more adherence to dietary, exercise and doctor's recommendations. There was no relationship between marital quality and adherence to blood glucose testing and control. the level of marital quality wasn't predictor of adherence to different aspects diabetes self‐care
Enzlin et al.^13^	Cross‐sectional study *N* = 300	Belgium	Case with complication: Mean age of 34.4 (8.5) Case without complication: Mean age of 39.6 (11.3) Control: Mean age of 35.8 (9.4)	100% female	The quality of marital relation (The Dyadic Adjustment Scale [DAS])	Type 1 Diabetes	Women with type 1 diabetes in comparison with non‐diabetic woman: ↓ marital quality
Whisman et al.^26^	Retrospective cohort study over 2 years *N* = 3898	US	Male: mean age of 64.74 (9.43) Female: mean age of 63.51 (8.56)	Male 48.18% Female 51.82%	Positive and negative partner exchange: consist of Positive supportive interaction and Negative unsupportive interactions (two scales from the Midlife Development in the United States survey measure)	Prevalence of type 2 diabetes	Decreasing frequency of positive exchanges and increasing frequency of negative exchanges in men: ↑prevalence of diabetes. Gender acts as a moderator of the relationship between partner exchanges and diabetes status
Liu et al.^18^	Retrospective cohort study over 5 years *N* = 1228	US	Mean age of 54.05 in range 57−64 Mean age of 31.61 in range 65−74 Mean age of 14.34 in range 75−85	Male 62.13% Female 37.87%	Marital quality (an 8‐item scale)	Type 2 Diabetes risk; type 2 diabetes management	Increase of positive marital quality in women: ↓risk of diabetes in women increase of negative marital quality in men: ↓ risk of diabetes and better diabetes management in men
Roberson et al.^21^	Retrospective cohort study over 5 years *N* = 800	US	Mean age of 55 (11.54)	Male 49.9% Female 50.1%	Marital strain, marital support, marital risk (relationship trouble, separate risk) and Constructive communication	Type 2 Diabetes risk; type 2 diabetes management	↑Marital risk and strain: ↑risk of diabetes. ↑marital strain and poor communication: ↑risk of diabetes and ↓diabetes management ↑marital support in lower income group: ↓risk of diabetes
Fisher et al.^17^	Cross‐sectional study *N* = 158	US	Mean age 56.9 (7.8)	Male 58.86% Female 41.14%	Marital Satisfaction (a 13‐item scale developed by Burns)	Type 2 Diabetes management	↑Marital satisfaction: ↑diabetes management
Martire et al.^19^	Cross‐sectional study *N* = 129	US	Mean age of 66.05 (7.71)	Male 49.61% Female 50.39%	Marital tension and enjoyment (a question measuring the level of tension and enjoyment)	Type 2 diabetes symptoms	↑The marital tension in a day: increase in the severe symptoms of type 2 diabetes on that day
Pieper et al.^14^	Cross‐sectional study *N* = 40 (20 couples)	US	Mean age of 56	Male 50% Female 50%	Marital adjustment (Dyadic adjustment scale [DAS])	Beliefs about diabetes mellitus	Perceived barriers of following diet and taking medication by the diabetic subjects: ↑marital satisfaction and marital quality
Trief et al.^22^	Cross‐sectional study *N* = 78	US	Mean age of 45.8 (11.33)	Male 43.3% Female 57.7%	Marital adjustment (Spanier Marital Adjustment Scale [DAS]) Marital intimacy (Personal Assessment of Intimacy in Relationships [PAIR])	Glycemic control; diabetic quality of life; diabetic distress	↑Marital satisfaction: better diabetes related satisfaction, ↓ diabetic distress, better quality of life and ↓ impact from diabetes the relationship of marital adjustment and HbA1c, as predictor of glycemic control, were not significant
Dadgari et al.^16^	Cross‐sectional study *N* = 160	Iran	31.2% in range 41−50 6.8% in range 21−30	Male 35.62% Female 64.38%	Marital satisfaction (Enrich Marital Satisfaction Questionnaire)	Compatibility with type 2 diabetes	↑The marital satisfaction in women: ↑compatibility with type 2 diabetes in women. There isn't any significant correlation between marital satisfaction and aspects of compatibility among men and women
Trief et al.^25^	Prospective cohort study over 2 years *N* = 61	US	Mean age of 47.1 (11.6)	Male 37.7% Female 62.3%	Marital adjustment (Spanier Marital Adjustment Scale [DAS]) Marital intimacy (Personal Assessment of Intimacy in Relationships [PAIR])	Glycemic control; diabetic quality of life; diabetic distress; satisfaction of diabetic care regimen	↑Marital adjustment: ↓diabetic distress. ↑marital adjustment and perceived marital intimacy: ↑satisfaction with diabetes care regimen. Marital factors have no significant relationship with health‐related quality of life and glycemic control
Naqvi et al.^20^	Cross‐sectional study *N* = 200	US	Mean age of 53.41 (11.13)	Male 55% Female 45%	Relationship quality (5‐item Quality of Marriage Index [QMI])	Diabetes self‐care; Medication adherence; Diabetes self‐efficacy; Diabetes distress	↑Relationship quality: ↑diabetic self‐care and self‐efficacy. ↑marital satisfaction in black women: ↑medication adherence ↑relationship quality in black women and white men: ↑diabetic self‐efficacy
Schreiner‐Engel et al.^15^	Cross‐sectional study *N* = 123	US	Diabetic one: mean age of 34.2 Control one: mean age of 34.7 Diabetic two: mean age of 45.7 Control two: mean age of 45.7	100% female	Marital adjustment and satisfaction (the Locke‐Wallace Marital Inventory)	Type 1 diabetes; type 2 diabetes	Woman with type 2 diabetes, unlike type 1 diabetic subjects: ↓marital quality and satisfaction
Trief et al.^23^	Prospective cohort study over 1 year *N* = 134	US	Mean age of 70.11 (5.33)	Male 57.46% Female 42.54%	Marital adjustment (Spanier Dyadic Adjustment Scale [DAS]) Marital stress (Perceived marital stress scale [PMSS])	Blood glucose; diabetes related distress	↑Marital stress: ↑diabetes distress and ↓blood glucose control. ↑marital satisfaction: better blood glucose control

## 3 RESULTS

The preliminary search of three databases resulted in 189 articles. After removing duplicates, 103 studies remained, of which, 76 articles did not meet the inclusion/exclusion criteria, and 27 articles were kept for full‐text review by both authors. Full‐text review of the remaining articles resulted in the further exclusion of 10 articles based on the inclusion/exclusion criteria, and the full text of 3 articles was not available. Finally, 14 articles fulfilled the expected criteria. The included articles were divided into 2 general groups. The first group consisted of 3 articles^13^, ^14^, ^15^ examining the influence of diabetes mellitus factors on the level of marital quality, and the second group included 11 articles^16^, ^17^, ^18^, ^19^, ^20^, ^21^, ^22^, ^23^, ^24^, ^25^, ^26^ studying the impact of marital quality status on diabetes‐related factors. In the following, we will first present the studies of the first group, and then the studies related to the second group will be presented. Table 3 summarizes the characteristics and significant results of included studies.

### 3.1 Impact of diabetes‐related factors on marital quality

The studies in this section fall into two categories. The first group directly investigated the effect of diabetes on marital quality and the other group investigated the effect of beliefs related to diabetes on marital quality.

#### 3.1.1 Direct effect of diabetes mellitus on marital quality

A cross‐sectional study on women with type 1 diabetes by Enzlin et al. showed that women with diabetes have had lower marital quality than the subjects without diabetes.^13^ Moreover, results of another cross‐sectional study on women with diabetes by Schreiner‐Engel et al. showed that, unlike type 1 diabetes, type 2 diabetes has had a negative influence on marital satisfaction in women.^15^

#### 3.1.2 Effect of beliefs about diabetes on marital quality

Beliefs about diabetes are defined as the factors of controlling diabetes, barriers and social supports of following diets, barriers of using drugs, the influence of job on the procedure of treatment, and adherence to the advantages of treatment.^14^ A cross‐sectional study by Pieper et al. found that perceived barriers to diet and medication adherence by subjects with diabetes are linked to higher marital satisfaction and marital quality.^14^

### 3.2 Role of marital quality in diabetes‐related factors

The studies in this section have generally investigated the impact of marital quality on four areas related to diabetes including the risk of developing diabetes, diabetes management, quality of life in patients with diabetes, and adherence to diabetes care regimen, which are presented in order.

#### 3.2.1 Risk of developing diabetes

Several studies investigated marital quality as a risk factor for type 2 diabetes. A 5‐year retrospective cohort study by Roberson et al. found that marital risk and strain were associated with the risk of having diabetes. Furthermore, marital strain and poor communication have had an association with an increase in the risk of developing diabetes. In low‐income subjects, there is an inverse relationship between marital support and the risk of diabetes.^21^ Another retrospective cohort study over 5 years by Liu et al. suggested that the increase in positive marital quality results in a lower risk of diabetes in women. Unexpectedly, an increase in negative marital quality is associated with a lower risk of diabetes in men.^18^ A 2‐year retrospective cohort study by Whisman et al. found that decreasing frequency of positive exchanges and increasing frequency of negative exchanges between spouses were linked to a higher prevalence of diabetes in men. Whereas, this association was not found in women. Also, the relationship between partner exchanges and diabetes status was moderated by gender.^26^

#### 3.2.2 Diabetes management

A 1‐year prospective cohort study by Trief et al. revealed that higher marital stress was associated with poor blood glucose control at the initial measurement of the study. Also, higher marital satisfaction is a predictor of better blood glucose control.^23^ A 5‐year retrospective cohort study by Roberson et al. found that marital strain and poor communication have a significant relationship with a lower level of diabetes management.^21^ Surprisingly, the results of a retrospective cohort study by Liu et al. showed that in men, higher negative marital quality results in better diabetes management.^18^ Another cross‐sectional study by Fisher et al. found that there is an obvious association between marital satisfaction and diabetes management.^17^

A cross‐sectional study by Naqvi et al. examined the impact of marital quality on diabetes‐related self‐care (including medication adherence, checking blood glucose, exercise, and dietary intake) and self‐efficacy, defined as the confidence in controlling diabetes. Results suggested that relationship quality is linked to higher self‐care and self‐efficacy. In addition, there is a significant correlation between relationship quality and self‐efficacy among black women and white men.^20^ In contrast, some studies yielded conflicting results. A cross‐sectional study by Trief et al. showed that the relationship between marital adjustment and HbA1c, as a predictor of glycemic control, was not noteworthy.^22^ Also, another 2‐year prospective cohort study by Trief et al. suggested that there is no significant association between marital factors and glycemic control.^25^

#### 3.2.3 Quality of life in patients with diabetes

A cross‐sectional study by Trief et al. revealed that higher marital satisfaction is associated with better diabetes‐related satisfaction, lower diabetes‐related distress, a better quality of life, and lower impact from diabetes.^22^ Furthermore, another 2‐year prospective cohort study by Trief et al. found that higher levels of marital adjustment, result in lower diabetes‐related distress. Also, higher marital adjustment and perceived marital intimacy were predictors of satisfaction with the diabetes care regimen. Hence, marital adjustment and marital intimacy were associated with aspects of health‐related quality of life. Nonetheless, there wasn't any significant relationship between marital adjustment and intimacy with general health‐related quality of life.^25^ Moreover, results of a 1‐year prospective cohort study by Trief et al. proved the significant direct association between marital stress and diabetes distress.^23^

A cross‐sectional study by Dadgari et al. suggested that marital satisfaction has a significant association with compatibility with type 2 diabetes in women. Although, this result is not valid for men. This study also found that there is not any significant correlation between marital satisfaction and aspects of compatibility among men and women, including attitude toward diseases, dependency and independency conflict, relationship with friends, family relationship, and physical image.^16^

The relationship between marital quality and symptoms of diabetes was discussed in a cross‐sectional study on older adults by Martire et al. which revealed that daily marital tension can increase the severe symptoms of type 2 diabetes mellitus on that day.^19^

#### 3.2.4 Adherence to diabetes care regimen

The association between marital quality and adherence to diabetes treatment was examined in several studies. Results of a prospective cohort study over 2 years by Trief et al. suggested that marital quality and intimacy were associated with adherence to dietary, exercise, and doctor's recommendations at the beginning of the study. Whereas, there was no relationship between marital quality and adherence to blood glucose testing and control. Also, the initial level of marital quality was not a predictor of adherence to different aspects of diabetes self‐care at the end of the study.^24^ A cross‐sectional study by Naqvi et al. found that in black women, marital satisfaction has had an obvious link with medication adherence.^20^

## 4 DISCUSSION

The fact of worldwide increasing prevalence of diabetes mellitus and the burden of its complications in patients with diabetes^29^ has amplified the interest of researchers in investigating its association with other health‐related determinants, especially psychological factors. This systematic review is the first one to examine the association between marital quality and diabetes mellitus. The studies investigating this association were divided into two main study fields. The first is about the effect of diabetes‐related factors on marital quality and the other is about the influence of marital quality on multiple diabetes‐related factors.

As the main determinant of physical and psychological health status,^30^ the influence of diabetes on marital quality was discussed in three studies. A study by Enzlin et al. indicated the negative effect of type 1 diabetes on the quality of marital relationships in women.^13^ As this article stated, the group with diabetes consisted of women with and without diabetes‐related complications. Thus, the effect of complications related to diabetes on marital quality should have been considered in the comparison of groups with and without diabetes. The other cross‐sectional study did not indicate the same results stated by the previous article and showed the significant effect of type 2 diabetes on marital satisfaction.^15^ Despite studying the relationship only in the female group, its results are more noteworthy since it has studied both patients with diabetes mellitus type 1 and 2. A cross‐sectional study examined a different aspect of diabetes and stated the benefits of beliefs about diabetes on marital quality.^14^ But the sample size of this study was small, which may not represent the subjects with diabetes and increases the risk of selection bias. Also, the gender‐related impacts were not discussed in this study. Overall, these studies show the adverse effect of diabetes on marital quality and the advantages of beliefs about diabetes on marital quality. However, the opposite results, which have caused doubts about the validity of the mentioned results (due to the limited participants and gender groups), show the necessity of examining this relationship in further studies. Also, the impact of different aspects of diabetes, such as diabetes management or adherence to diabetic care on marital quality and its dimensions, demands more attention from researchers in other studies.

In addition, the articles which studied the impact of marital quality on diabetes mellitus can be discussed in several fields. Some studies considered marital quality as an important factor in diabetes risk.^18^, ^21^, ^26^ The retrospective cohort study by Roberson et al.^21^ was based on the national midlife in the United States (MIDUS) data set^31^, ^32^ and focused on both positive (marital support and constructive communication) and negative (marital risk and strain) aspects of marriage. Also, this study examined the moderator role of sociodemographic determinants like income in this association. The other retrospective cohort^18^ used data from the National Social Life, Health, and Aging Project in the United States (NSHAP).^33^, ^34^ This study also measured both positive and negative marital quality and the data were analyzed by gender. But the challenging result of this study is the effect of marital quality on men. Liu et al. found that negative marital quality acts as a preventative factor for diabetes onset, which is clearly different from the findings of other researchers. The last study about the risk of diabetes is the retrospective cohort study by Whisman et al.^26^ This study was based on the data of the health and retirement study^35^ in the United States and studied the effects of positive and negative exchanges with spouses on diabetes onset both in men and women. This research had fewer follow‐up years than previous studies. In summary, almost all of these retrospective studies stated the preventative effect of positive marital quality and the adverse impact of negative aspects of marital quality on the onset of type 2 diabetes. Nevertheless, some contradictory results on this association, especially in men, need to be clarified in future research.

Diabetes mellitus requires lifelong management to prevent other complications of this metabolic disease such as neuropathy and nephropathy.^36^ Numerous studies examined the effect of marital relationship quality on diabetes management, and their consensus was on the positive association between these factors.^17^, ^18^, ^20^, ^21^, ^23^ Fisher et al. showed a direct link between relationship quality and diabetes management.^17^ This study has some biases including a 47% acceptance rate that shows losing about half of the study sample. Also, the study by Liu et al.^18^ requires more investigation due to its challenging results of the direct association between negative marital quality and diabetes management in men. The other cross‐sectional study by Naqvi et al.^20^ investigated the role of sex, race, and relationship quality in diabetes‐related self‐care and self‐efficacy. Considering the role of age and gender in this association may make its results more worthwhile. The other study investigating the association between different aspects of marital quality and diabetes management was by Roberson et al.^21^ As stated previously, its results were more noteworthy, since they included different socioeconomic factors in their analyses. The prospective cohort study over 1 year by Trief et al.^23^ was conducted on the participants of the Informatics for Diabetes Education and Telemedicine Project (IDEATel). However, less number of participants and years of follow‐up may attenuate the results. In addition, some studies have rejected this positive association with glycemic control.^22^, ^25^ Trief et al. in a cross‐sectional study^22^ conclude that there isn't any significant association between marital adjustment and glycemic control. Despite its satisfactory method, the strength of results may decrease due to the small sample size. The next prospective cohort study by Trief et al.^25^ was based on the sample of a previous cross‐sectional study.^22^ This study also rejected the predictor role of marital measures on glycemic control. But like the previous study, the small size sample challenges the stated results. Also, as this study examined the influence of a psychological factor on diabetes, more years of follow‐up are needed.

The effect of marital quality on various aspects of quality of life in patients with diabetes, including diabetes‐related distress, satisfaction of diabetic regimen, compatibility with diabetes, symptom severity, diabetes‐related satisfaction, and overall life quality, was another diabetes‐related outcome examined in the studies. The main agreement of the results of these studies was on the positive effects of marital quality on different aspects of the lives of subjects with diabetes, except the general life quality of subjects.^16^, ^22^, ^23^, ^25^ A study^22^ stated the positive impact of marital satisfaction and intimacy on various aspects of the lives of patients with diabetes, including diabetic satisfaction, lower diabetic distress, and general quality of life. Also, the other study by Trief et al.^25^ based on the previous cross‐sectional study, agreed with the previous results on various aspects of quality of life, except the impact of marital adjustment and intimacy on general health‐related quality of life, which may need to be more discussed in other studies with more participants and years of follow‐up. Trief et al. by conducting a cohort study^23^ suggested the predicting role of marital stress for diabetes distress. However, because of the reasons stated before, this study may not have as strong results as the others. A cross‐sectional study^16^ showed the association between marital quality and diabetic compatibility and its different aspects based on gender. But its conflicting results for overall compatibility and aspects of compatibility need to be clarified in other studies with more participants. Also, Martire et al. found that high marital tension in a day worsens the severe symptoms of diabetes.^19^ However, this study may potentially have selection bias and needs to determine this association based on age and gender. In conclusion, marital quality has an influence on different aspects of quality of life in subjects with diabetes, including diabetic distress, satisfaction with the diabetic regimen, compatibility with diabetes, symptom severity, diabetes‐related satisfaction, and overall life quality.

Diabetes is a life‐long disease that needs to have an active adherence to the diabetic care regimen such as exercise and controlling blood glucose to contribute the diabetes management.^37^ Given the importance of adherence in the treatment and controlling of diabetes mellitus,^38^ adherence to the diabetic care regimen was the main subject discussed by several articles. Trief et al. in a cohort study^24^ showed the effect of marital intimacy and adjustment on adherence to various aspects of diabetic self‐care at the beginning of the study. However, the predictor role of marital factors was not confirmed. This study had some limitations that may attenuate its results, including the restricted number of participants and years of follow‐up. In a cross‐sectional study, Naqvi et al.^20^ confirmed this association based and gender and race, which may give more strength to the results.^20^ Overall, although some results^24^ have not confirmed some aspects of this relationship, other results^20^, ^24^ have highlighted the effect of marital quality on the adherence to diabetic care, which can be considered as a major determinant in treatment procedures.

As mentioned, this study, as the first systematic review, aims to investigate the relationship between diabetes and marital quality and the mutual influence of these two. Since this relationship has not been systematically examined before, it can be useful to review studies that have examined the relationship between marital quality and other chronic diseases (other than diabetes). Although the relationship between marital quality and chronic diseases has generally been neglected, below are two examples of such studies:

Bennett‐Britton et al. examined the association between marital relationship quality changes and risk factors related to cardiovascular disease in men. They found that low levels of low‐density lipoprotein are associated with better relationships. However, the association between total cholesterol and diastolic blood pressure and improved relationships was weaker. Higher diastolic blood pressure was associated with worsening relationships.^39^ In another study, Birditt et al. investigated the relationship between marital/partner, stress, quality, and blood pressure. Although spousal/partner stress or quality did not have the main effects on blood pressure, the relationship between stress and blood pressure was moderated by spousal/partner quality. The negative associations between stress and blood pressure were observed specifically among the individuals reporting less reliance, more confiding, and greater demands from their spouses/partners.^40^ Therefore, as can be seen, marital quality has significant effects on chronic diseases.

Finally, to summarize the results of this study, the following section is given:

The studies were divided into two general groups. The first group consisted of three articles examining the effect of factors related to diabetes on marital quality, and the second group included 11 articles studying the effect of marital quality on diabetes and its factors. In general, the articles investigating the impact of diabetes‐related factors on marital quality showed that diabetes has negative impacts on levels of marital quality. These impacts can be a direct effect of diabetes or they can be through beliefs about diabetes. For example, perceived barriers to diet and medication adherence are linked to higher marital satisfaction and marital quality. In the second part, which was studies that investigated the effect of marital quality on different aspects of diabetes, several results were obtained. Regarding the risk of developing diabetes, it was shown that marital risk and strain, poor communication, and decreasing frequency of positive exchanges were associated with an increase in the risk of developing diabetes. Regarding diabetes management, the results were conflicting so some of the studies have indicated that higher marital stress, marital strain, and poor communication were associated with a lower level of diabetes management such as poor blood glucose control. However, another group of studies also stated that marital quality did not affect diabetes management. Regarding the quality of life in patients with diabetes, it was shown that higher marital satisfaction and adjustment are associated with better diabetes‐related satisfaction, lower diabetic distress, a better quality of life, and lower impact from diabetes. However, several conflicting results were presented in this section. For example, although marital adjustment and perceived marital intimacy were predictors of satisfaction with the diabetes care regimen, however, they did not remarkably associate with general health‐related quality of life. Also, a study stated that there is not any significant correlation between marital satisfaction and aspects of compatibility among men and women, including attitude toward diseases, dependency and independency conflict, relationship with friends, family relationship, and physical image. Regarding adherence to the diabetes care regimen, it was mentioned in general that marital quality, satisfaction, and intimacy were associated with adherence to the regimen, however, a study stated that there was no relationship between marital quality and adherence to blood glucose testing and control. Therefore, it is important to investigate which adherence factors are affected.

In a number of investigated relationships, some studies have pointed to the moderating role of gender. Regarding the risk of developing diabetes, a study has pointed to the opposite results, so unlike the other results of this section, it has pointed out that an increase in negative marital quality is associated with a lower risk of diabetes in men. This study also pointed out the inverse relationship between marital quality and diabetes management in men. Also, another study investigating the risk of developing diabetes has mentioned that the condition of diabetes in women was independent of marital quality. Regarding the quality of life, a study has mentioned that marital satisfaction has a significant association with compatibility with type 2 diabetes in women, although, this result is not valid for men. Finally, in the section related to adherence to the regimen, the role of women's gender is mentioned. Considering these results, the importance of gender differences and their moderating role should be addressed in future studies.

## 5 LIMITATION

This study had serious limitations. The most important limitation is the lack of related articles. Unfortunately, the role of psychological factors, especially marital quality, in chronic diseases and vice versa has been neglected for many years, and there is no suitable comprehensive keyword for marital quality. In the results section, the studies are classified into six sub‐titles, each of which can be the subject of an article. But we believed that detailing the topic of this article and making it more precise, although it would make the study more specialized, due to the general neglect of this relationship by researchers, could not be a proper guide for subsequent studies. Also, by narrowing the topic, the number of articles (which is still few) would be significantly reduced and their results would not be suitable for independent presentation. In this situation, the present study tried to investigate the relationship between diabetes and marital quality in a broad way to prepare the ground for further studies in this field in a more targeted and detailed manner. The current systematic review tries to provide a general summary of the studies conducted in the past 30 years and guide the new studies under the six sub‐titles introduced.

It is natural that due to the small number of studies, the results presented in each of the subtitles are not strong enough, especially since the studies themselves had serious limitations that were presented in the discussion section. Also, some articles could be presented in more than one subtitle.

In addition, owing to the limitation of studies, variation in data collection, methods of marital quality measurement, and studied population, conducting a meta‐analysis was not possible. Future studies can provide the opportunity of conducting meta‐analysis and more accurate and reliable conclusions by using the results of this systematic review and not repeating the biases raised for the included studies in the discussion section.

## 6 CONCLUSION

Based on this systematic review, diabetes mellitus acts as an important influencer on the level of marital quality. Despite some conflicting results, this impact was concluded by several studies and should be considered as one of the diabetes outcomes which influence the life quality of patients with diabetes. Also, the level of quality of the marital relationship may determine various factors related to type 2 diabetes, including the risk of having diabetes, disease management, life quality, and adherence to the diabetic care regimen. In most cases, an increase in marital quality leads to the improvement of various health‐related aspects of subjects with diabetes. Therefore, in further studies and medical interventions performed for patients with diabetes, the role of marital quality which has an important impact on the quality of life should be considered. However, since there are some conflicting and weak results, this association and its mechanism of action require more attention from researchers in future studies.

## AUTHOR CONTRIBUTIONS

**Mohsen Rastkar**: Conceptualization; data curation; methodology; project administration; resources; supervision; validation; visualization; writing – original draft; writing – review and editing. **Erfan Jalalifar**: Conceptualization; data curation; methodology; project administration; resources; supervision; validation; visualization; writing – original draft; writing – review and editing. All authors have read and approved the final version of the manuscript.

## CONFLICT OF INTERESTS STATEMENT

The authors declare no conflict of interest.

## TRANSPARENCY STATEMENT

The lead author Erfan Jalalifar affirms that this manuscript is an honest, accurate, and transparent account of the study being reported; that no important aspects of the study have been omitted; and that any discrepancies from the study as planned (and, if relevant, registered) have been explained.

## Supporting information

Supplementary information.

## Data Availability

The authors confirm that the data supporting the findings of this study are available within the article [and/or] its Supporting Information Materials. E. J. had full access to all of the data in this study and takes complete responsibility for the integrity of the data and the accuracy of the data analysis.

## References

[1] Fincham FD , Bradbury TN . The assessment of marital quality: a reevaluation. J Marriage Fam. 1987;49:797‐809.

[2] Delatorre M , Wagner A . Marital quality assessment: reviewing the concept, instruments, and methods. Marri Fam Rev. 2020;56:1‐24.

[3] Burman B , Margolin G . Analysis of the association between marital relationships and health problems: an interactional perspective. Psychol Bull. 1992;112:39‐63. 1529039 10.1037/0033-2909.112.1.39

[4] Robles TF , Slatcher RB , Trombello JM , Mcginn MM . Marital quality and health: a meta‐analytic review. Psychol Bull. 2014;140:140‐187. 23527470 10.1037/a0031859 PMC3872512

[5] Atlas D . International diabetes federation. IDF Diabetes Atlas. *7th edn* . International Diabetes Federation; 2015.

[6] Zimmet P , Alberti KG , Magliano DJ , Bennett PH . Diabetes mellitus statistics on prevalence and mortality: facts and fallacies. Nat Rev Endocrinol. 2016;12:616‐622. 27388988 10.1038/nrendo.2016.105

[7] Diagnosis and classification of diabetes mellitus. Diabetes Care. 2013;36(suppl 1):S67‐S74. 10.2337/dc13-S067 23264425 PMC3537273

[8] Semenkovich K , Brown ME , Svrakic DM , Lustman PJ . Depression in type 2 diabetes mellitus: prevalence, impact, and treatment. Drugs. 2015;75:577‐587. 25851098 10.1007/s40265-015-0347-4

[9] Barlow J , Wright C , Sheasby J , Turner A , Hainsworth J . Self‐management approaches for people with chronic conditions: a review. Patient Educ Couns. 2002;48:177‐187. 12401421 10.1016/s0738-3991(02)00032-0

[10] Clari M , Matarese M , Ivziku D , De Marinis MG . Self‐care of people with chronic obstructive pulmonary disease: a meta‐synthesis. Patient. 2017;10:407‐427. 28197788 10.1007/s40271-017-0218-z

[11] Maffi P , Secchi A . The burden of diabetes: emerging data. Dev Ophthalmol. 2017;60:1‐5. 28427059 10.1159/000459641

[12] Assar ME , Laosa O , Rodríguez Mañas L . Diabetes and frailty. Curr Opi Clin Nutri Meta Care. 2019;22:52‐57. 10.1097/MCO.0000000000000535 30394893

[13] Enzlin P , Mathieu C , Van den Bruel A , Bosteels J , Vanderschueren D , Demyttenaere K . Sexual dysfunction in women with type 1 diabetes. Diabetes Care. 2002;25:672‐677. 11919123 10.2337/diacare.25.4.672

[14] Pieper BA , Kushion W , Gaida S . The relationship between a couple's marital adjustment and beliefs about diabetes mellitus. Diabetes Educ. 1990;16:108‐112. 2311501 10.1177/014572179001600206

[15] Schreiner‐Engel P , Schiavi RC , Vietorisz D , Smith H . The differential impact of diabetes type on female sexuality. J Psychosom Res. 1987b;31:23‐33. 3820143 10.1016/0022-3999(87)90094-8

[16] Dadgari A , Mazloom N , Heidari Firouz Abadi MR , Bagheri I . The relationship between marital satisfaction and compatibility with type 2 diabetes. Iran J Psychiatry Behav Sci. 2015;9:9. 10.17795/ijpbs-3105 PMC4733311 26834807

[17] Fisher L , Chesla CA , Chun KM , et al. Patient‐appraised couple emotion management and disease management among Chinese American patients with type 2 diabetes. J Fam Psychol. 2004;18:302‐310. 15222837 10.1037/0893-3200.18.2.302

[18] Liu H , Waite L , Shen S . Diabetes risk and disease management in later life: a national longitudinal study of the role of marital quality. J Gerontol B Psychol Sci Soc Sci. 2016;71:1070‐1080. 27216861 10.1093/geronb/gbw061 PMC5067945

[19] Martire LM , Hemphill RC , Zhaoyang R , Stephens MAP , Franks MM , Stanford AM . Daily marital tension and symptom severity in older adults with diabetes or osteoarthritis. Ann Behav Med. 2018;52:842‐853. 29579164 10.1093/abm/kax062 PMC6135959

[20] Naqvi JB , Helgeson VS , Gary‐Webb TL , Korytkowski MT , Seltman HJ . Sex, race, and the role of relationships in diabetes health: intersectionality matters. J Behav Med. 2020;43:69‐79. 31102104 10.1007/s10865-019-00057-w PMC6858941

[21] Roberson PNE , Fincham F . Is relationship quality linked to diabetes risk and management? It depends on what you look at. Fam Syst Health. 2018;36:315‐326. 29999343 10.1037/fsh0000336

[22] Trief PM , Himes CL , Orendorff R , Weinstock RS . The marital relationship and psychosocial adaptation and glycemic control of individuals with diabetes. Diabetes Care. 2001;24:1384‐1389. 11473074 10.2337/diacare.24.8.1384

[23] Trief PM , Morin PC , Izquierdo R , et al. Marital quality and diabetes outcomes: the IDEATel project. Fam Syst Health. 2006;24:318‐331.

[24] Trief PM , Ploutz‐Snyder R , Britton KD , Weinstock RS . The relationship between marital quality and adherence to the diabetes care regimen. Ann Behav Med. 2004;27:148‐154. 15184090 10.1207/s15324796abm2703_2

[25] Trief PM , Wade MJ , Britton KD , Weinstock RS . A prospective analysis of marital relationship factors and quality of life in diabetes. Diabetes Care. 2002;25:1154‐1158. 12087013 10.2337/diacare.25.7.1154

[26] Whisman MA , Li A , Sbarra DA , Raison CL . Marital quality and diabetes: results from the health and retirement study. Health Psychol. 2014;33:832‐840. 25068454 10.1037/hea0000064

[27] Stang A . Critical evaluation of the Newcastle‐Ottawa scale for the assessment of the quality of nonrandomized studies in meta‐analyses. Eur J Epidemiol. 2010;25:603‐605. 20652370 10.1007/s10654-010-9491-z

[28] Liberati A , Altman DG , Tetzlaff J , et al. The PRISMA statement for reporting systematic reviews and Meta‐Analyses of studies that evaluate health care interventions: explanation and elaboration. JCE. 2009;62:e1‐e34. 19631507 10.1016/j.jclinepi.2009.06.006

[29] Koye DN , Magliano DJ , Nelson RG , Pavkov ME . The global epidemiology of diabetes and kidney disease. Adv Chronic Kidney Dis. 2018;25:121‐132. 29580576 10.1053/j.ackd.2017.10.011 PMC11000253

[30] Robles TF . Marital quality and health: implications for marriage in the 21(st) century. Curr Dire Psychol Sci. 2014;23:427‐432. 10.1177/0963721414549043 PMC4275835 25544806

[31] Ryff C , Almeida DM , Ayanian J , et al. Midlife in the United States (MIDUS 2), 2004‐2006. Inter‐university consortium for political and social research [distributor]. 2017.

[32] Ryff CD , Seeman T , Weinstein M . Midlife in the United States (MIDUS 2): Biomarker Project, 2004‐2009. Inter‐university consortium for political and social research [distributor]. 2019.

[33] Waite LJ , Cagney KA , Dale W , et al. National Social Life, Health, and Aging Project (NSHAP): Wave 2 and Partner Data Collection, [United States], 2010‐2011. Inter‐university consortium for political and social research [distributor]. 2019.

[34] Waite LJ , Laumann EO , Levinson WS , Lindau ST , O'muircheartaigh CA . National Social Life, Health, and Aging Project (NSHAP): Wave 1, [United States], 2005‐2006. Inter‐university consortium for political and social research [distributor]. 2019.

[35] Fisher GG , Ryan LH . Overview of the health and retirement study and introduction to the special issue. Work Agi Retire. 2018;4:1‐9. 10.1093/workar/wax032 PMC5798643 29423243

[36] Nam S , Chesla C , Stotts NA , Kroon L , Janson SL . Barriers to diabetes management: patient and provider factors. Diabetes Res Clin Pract. 2011;93:1‐9. 21382643 10.1016/j.diabres.2011.02.002

[37] Cramer JA . A systematic review of adherence with medications for diabetes. Diabetes Care. 2004;27:1218‐1224. 15111553 10.2337/diacare.27.5.1218

[38] Lutfey KE , Wishner WJ . Beyond ‘compliance’ is ‘adherence’: improving the prospect of diabetes care. Diabetes Care. 1999;22:635‐639. 10189544 10.2337/diacare.22.4.635

[39] Bennett‐Britton I , Teyhan A , Macleod J , Sattar N , Davey Smith G , Ben‐Shlomo Y . Changes in marital quality over 6 years and its association with cardiovascular disease risk factors in men: findings from the ALSPAC prospective cohort study. J Epidemiol Community Health. 2017;71:1094‐1100. 28993473 10.1136/jech-2017-209178 PMC5847094

[40] Birditt KS , Newton N , Hope S . Implications of marital/partner relationship quality and perceived stress for blood pressure among older adults. J Gerontol B Psychol Sci Soc Sci. 2014;69:188‐198. 23275499 10.1093/geronb/gbs123 PMC3976116

