# Supplementary material for: The association between marital quality and diabetes mellitus: A systematic review
Source: Health Sci Rep. 2023 Feb 7;6(2):e1106. doi: 10.1002/hsr2.1106 (PMC9905793; doi:10.1002/hsr2.1106)
Supplement: Supplementary file 1 — Supplementary information. [file HSR2-6-e1106-s001.docx]

Supplementary material: search strategy

| Database | Date | Search Strategy |
| --- | --- | --- |
| Medline | 9.4.2021 | (((((((((((((marital quality[Text Word]) OR (marital satisfaction[Text Word])) OR (Marital stability[Text Word])) OR (relationship stability[Text Word])) OR (Marital interaction quality[Text Word])) OR (Spousal relationship quality[Text Word])) OR (Marital adjustment[Text Word])) OR (Marital tension[Text Word])) OR (Marital support[Text Word])) OR (marital intimacy[Text Word])) OR (marital stress[Text Word])) OR (marital cohesion[Text Word])) OR (Marital strain[Text Word)) AND (((((((((((((((((((((((((((((((((((((((((((((((((((((((((((((((((diabetes mellitus[MeSH Terms]) OR (diabetes mellitus[Text Word])) OR (Diabetes Mellitus, Type 2[Mesh Terms])) OR (Diabetes Mellitus, Type 2[Text Word])) OR (Diabetes Mellitus, Noninsulin-Dependent[Text Word])) OR (Diabetes Mellitus, Ketosis-Resistant[Text Word])) OR (Diabetes Mellitus, Ketosis Resistant[Text Word])) OR (Ketosis-Resistant Diabetes Mellitus[Text Word])) OR (Diabetes Mellitus, Non Insulin Dependent[Text Word])) OR (Diabetes Mellitus, Non-Insulin-Dependent[Text Word])) OR (Non-Insulin-Dependent Diabetes Mellitus[Text Word])) OR (Diabetes Mellitus, Stable[Text Word])) OR (Stable Diabetes Mellitus[Text Word])) OR (Diabetes Mellitus, Type II[Text Word])) OR (NIDDM[Text Word])) OR (Diabetes Mellitus, Noninsulin Dependent[Text Word])) OR (Diabetes Mellitus, Maturity-Onset[Text Word])) OR (Diabetes Mellitus, Maturity Onset[Text Word])) OR (Maturity-Onset Diabetes Mellitus[Text Word])) OR (Maturity Onset Diabetes Mellitus[Text Word])) OR (MODY[Text Word])) OR (Diabetes Mellitus, Slow-Onset[Text Word])) OR (Diabetes Mellitus, Slow Onset[Text Word])) OR (Slow-Onset Diabetes Mellitus[Text Word])) OR (Type 2 Diabetes Mellitus[Text Word])) OR (Noninsulin-Dependent Diabetes Mellitus[Text Word])) OR (Noninsulin Dependent Diabetes Mellitus[Text Word])) OR (Maturity-Onset Diabetes[Text Word])) OR (Diabetes, Maturity-Onset[Text Word])) OR (Maturity Onset Diabetes[Text Word])) OR (Type 2 Diabetes[Text Word])) OR (Diabetes, Type 2[Text Word])) OR (Diabetes Mellitus, Adult-Onset[Text Word])) OR (Adult-Onset Diabetes Mellitus[Text Word])) OR (Diabetes Mellitus, Adult Onset[Text Word])) OR (Diabetes Mellitus, Type 1[MeSH Terms])) OR (Diabetes Mellitus, Type 1[MeSH Terms])) OR (Diabetes Mellitus, Insulin-Dependent[Text Word])) OR (Diabetes Mellitus, Insulin Dependent[Text Word])) OR (Insulin-Dependent Diabetes Mellitus[Text Word])) OR (Diabetes Mellitus, Juvenile-Onset[Text Word])) OR (Diabetes Mellitus, Juvenile Onset[Text Word])) OR (Juvenile-Onset Diabetes Mellitus[Text Word])) OR (IDDM[Text Word])) OR (Juvenile-Onset Diabetes[Text Word])) OR (Diabetes, Juvenile-Onset[Text Word])) OR (Juvenile Onset Diabetes[Text Word])) OR (Diabetes Mellitus, Sudden-Onset[Text Word])) OR (Diabetes Mellitus, Sudden Onset[Text Word])) OR (Sudden-Onset Diabetes Mellitus[Text Word])) OR (Type 1 Diabetes Mellitus[Text Word])) OR (Diabetes Mellitus, Insulin-Dependent, 1[Text Word])) OR (Insulin-Dependent Diabetes Mellitus 1[Text Word])) OR (Insulin Dependent Diabetes Mellitus 1[Text Word])) OR (Type 1 Diabetes[Text Word])) OR (Diabetes, Type 1[Text Word])) OR (Diabetes Mellitus, Type I[Text Word])) OR (Diabetes, Autoimmune[Text Word])) OR (Autoimmune Diabetes[Text Word])) OR (Diabetes Mellitus, Brittle[Text Word])) OR (Brittle Diabetes Mellitus[Text Word])) OR (Diabetes Mellitus, Ketosis-Prone[Text Word])) OR (Diabetes Mellitus, Ketosis Prone[Text Word])) OR (Ketosis-Prone Diabetes Mellitus[Text Word])) OR (diabetes[Text Word])) |
| Scopus | 9.4.2021 | TITLE-ABS-KEY(("marital quality") OR ("marital satisfaction") OR ("Marital stability") OR ("relationship stability") OR ("Marital interaction quality") OR ("Spousal relationship quality") OR ("Marital adjustment") OR ("Marital tension") OR ("Marital support") OR ("marital intimacy") OR ("marital stress") OR ("marital cohesion") OR ("Marital strain")) AND TITLE-ABS-KEY(("diabetes mellitus") OR (diabetes) OR (Diabetes Mellitus, Type 2) OR (Diabetes Mellitus, Noninsulin-Dependent) OR (Diabetes Mellitus, Ketosis-Resistant) OR (Diabetes Mellitus, Ketosis Resistant) OR (Ketosis-Resistant Diabetes Mellitus) OR (Diabetes Mellitus, Non Insulin Dependent) OR (Diabetes Mellitus, Non-Insulin-Dependent) OR (Non-Insulin-Dependent Diabetes Mellitus) OR (Diabetes Mellitus, Stable) OR (Stable Diabetes Mellitus) OR (Diabetes Mellitus, Type II) OR (NIDDM) OR (Diabetes Mellitus, Noninsulin Dependent) OR (Diabetes Mellitus, Maturity-Onset) OR (Diabetes Mellitus, Maturity Onset) OR (Maturity-Onset Diabetes Mellitus) OR (Maturity Onset Diabetes Mellitus) OR (MODY) OR (Diabetes Mellitus, Slow-Onset) OR (Diabetes Mellitus, Slow Onset) OR (Slow-Onset Diabetes Mellitus) OR (Type 2 Diabetes Mellitus) OR (Noninsulin-Dependent Diabetes Mellitus) OR (Noninsulin Dependent Diabetes Mellitus) OR (Maturity-Onset Diabetes) OR (Diabetes, Maturity-Onset) OR (Maturity Onset Diabetes) OR (Type 2 Diabetes) OR (Diabetes, Type 2) OR (Diabetes Mellitus, Adult-Onset) OR (Adult-Onset Diabetes Mellitus) OR (Diabetes Mellitus, Adult Onset) OR (Diabetes Mellitus, Type 1) OR (Diabetes Mellitus, Insulin-Dependent) OR (Diabetes Mellitus, Insulin Dependent) OR (Insulin-Dependent Diabetes Mellitus) OR (Diabetes Mellitus, Juvenile-Onset) OR (Diabetes Mellitus, Juvenile Onset) OR (Juvenile-Onset Diabetes Mellitus) OR (IDDM) OR (Juvenile-Onset Diabetes) OR (Diabetes, Juvenile-Onset) OR (Juvenile Onset Diabetes) OR (Diabetes Mellitus, Sudden-Onset) OR (Diabetes Mellitus, Sudden Onset) OR (Sudden-Onset Diabetes Mellitus) OR (Type 1 Diabetes Mellitus) OR (Diabetes Mellitus, Insulin-Dependent, 1) OR (Insulin-Dependent Diabetes Mellitus 1) OR (Insulin Dependent Diabetes Mellitus 1) OR (Type 1 Diabetes) OR (Diabetes, Type 1) OR (Diabetes Mellitus, Type I) OR (Diabetes, Autoimmune) OR (Autoimmune Diabetes) OR (Diabetes Mellitus, Brittle) OR (Brittle Diabetes Mellitus) OR (Diabetes Mellitus, Ketosis-Prone) OR (Diabetes Mellitus, Ketosis Prone) OR (Ketosis-Prone Diabetes Mellitus)) |
| Web of Science | 9.4.2021 | TOPIC: ((("marital quality") OR ("marital satisfaction") OR ("Marital stability") OR ("relationship stability") OR ("Marital interaction quality") OR ("Spousal relationship quality") OR ("Marital adjustment") OR ("Marital tension") OR ("Marital support") OR ("marital intimacy") OR ("marital stress") OR ("marital cohesion") OR ("Marital strain"))) AND TOPIC: ((("diabetes mellitus") OR (diabetes) OR (Diabetes Mellitus, Type 2) OR (Diabetes Mellitus, Noninsulin-Dependent) OR (Diabetes Mellitus, Ketosis-Resistant) OR (Diabetes Mellitus, Ketosis Resistant) OR (Ketosis-Resistant Diabetes Mellitus) OR (Diabetes Mellitus, Non Insulin Dependent) OR (Diabetes Mellitus, Non-Insulin-Dependent) OR (Non-Insulin-Dependent Diabetes Mellitus) OR (Diabetes Mellitus, Stable) OR (Stable Diabetes Mellitus) OR (Diabetes Mellitus, Type II) OR (NIDDM) OR (Diabetes Mellitus, Noninsulin Dependent) OR (Diabetes Mellitus, Maturity-Onset) OR (Diabetes Mellitus, Maturity Onset) OR (Maturity-Onset Diabetes Mellitus) OR (Maturity Onset Diabetes Mellitus) OR (MODY) OR (Diabetes Mellitus, Slow-Onset) OR (Diabetes Mellitus, Slow Onset) OR (Slow-Onset Diabetes Mellitus) OR (Type 2 Diabetes Mellitus) OR (Noninsulin-Dependent Diabetes Mellitus) OR (Noninsulin Dependent Diabetes Mellitus) OR (Maturity-Onset Diabetes) OR (Diabetes, Maturity-Onset) OR (Maturity Onset Diabetes) OR (Type 2 Diabetes) OR (Diabetes, Type 2) OR (Diabetes Mellitus, Adult-Onset) OR (Adult-Onset Diabetes Mellitus) OR (Diabetes Mellitus, Adult Onset) OR (Diabetes Mellitus, Type 1) OR (Diabetes Mellitus, Insulin-Dependent) OR (Diabetes Mellitus, Insulin Dependent) OR (Insulin-Dependent Diabetes Mellitus) OR (Diabetes Mellitus, Juvenile-Onset) OR (Diabetes Mellitus, Juvenile Onset) OR (Juvenile-Onset Diabetes Mellitus) OR (IDDM) OR (Juvenile-Onset Diabetes) OR (Diabetes, Juvenile-Onset) OR (Juvenile Onset Diabetes) OR (Diabetes Mellitus, Sudden-Onset) OR (Diabetes Mellitus, Sudden Onset) OR (Sudden-Onset Diabetes Mellitus) OR (Type 1 Diabetes Mellitus) OR (Diabetes Mellitus, Insulin-Dependent, 1) OR (Insulin-Dependent Diabetes Mellitus 1) OR (Insulin Dependent Diabetes Mellitus 1) OR (Type 1 Diabetes) OR (Diabetes, Type 1) OR (Diabetes Mellitus, Type I) OR (Diabetes, Autoimmune) OR (Autoimmune Diabetes) OR (Diabetes Mellitus, Brittle) OR (Brittle Diabetes Mellitus) OR (Diabetes Mellitus, Ketosis-Prone) OR (Diabetes Mellitus, Ketosis Prone) OR (Ketosis-Prone Diabetes Mellitus))) |
